# Alkaloids in Processed Rhizoma Corydalis and Crude Rhizoma Corydalis Analyzed by GC/MS

**DOI:** 10.1155/2014/281342

**Published:** 2014-08-25

**Authors:** Zhifeng Guo, Ru Cai, Huidan Su, Yunlong Li

**Affiliations:** Key Laboratory of Medical Chemistry and Molecular Diagnosis, College of Chemistry and Environmental Science, Hebei University, Baoding 071002, China

## Abstract

The alkaloids in the processed Rhizoma Corydalis and the crude Rhizoma Corydalis were qualitatively and semiquantitatively analyzed using gas chromatography-mass spectrometry (GC/MS) method. The processing herb drug procedure was carried out according to the standard method of Chinese Pharmacopoeia. The samples were extracted using Soxhlet extractor with different solvents: methanol and acetone. The extraction effect on different solvents was investigated. The results showed that 11 kinds of alkaloids were identified from the crude Rhizoma Corydalis and only two were from the processed Rhizoma Corydalis. A total of 13 kinds of alkaloids were all based on two backbones. The alkaloids in the processed sample were less than those in the crude Rhizoma Corydalis significantly, while almost the corydaline has been changed in conformation after the sample had undergone processing, which provided support for the conclusion of reducing toxicity when the herbal medicine having been undergone a traditional drugs treatment process.

## 1. Introduction

Rhizoma Corydalis (RC), belonging to* Corydalis* species and the family of Papaveraceae, is a perennial herbaceous plant and grows mostly in Northeastern China. The tubers of RC have been employed as analgesic, sedative, and hypnagogue for long times [1, 2], which is a well-known traditional Chinese herbal medicine,* Corydalis yanhusuo* W.T. Wang and acts against myocardial ischemia, gastric ulcer, and tumour in the medical field. The bioactive constituents, corydaline, have analgesic efficacy but little toxicity [3–6].

The crude RC were used as samples in all phytochemical analysis, but the RC as traditional Chinese medicine often underwent processing. There is an old saying in China: “As a medicine, it is somewhat toxic,” so the folk pharmacists always make the natural herbal medicine a processing handcraft to enhance efficiency and reduce toxicity. It has been reported that more than 20 kinds of alkaloids are identified in RC [7–9], which have the similar biosynthetic route and backbone to berberine [10, 11]. These alkaloids are formed from two molecules of tyrosine. The tyrosines bioassemble the benzylisoquinolines, while the more immediate, pose the berberine basic structure [12]. The characteristics of these alkaloids are higher boiling points and lower vapor pressures. Thin-layer chromatography (TLC), high performance liquid chromatography (HPLC) [13–15], high performance capillary electrophoresis (HPLE) [16], and supercritical fluid chromatography [17] were often employed in the analysis of these alkaloids. Liquid chromatography-mass spectrometry (LC/MS) and multistage spectrometry (MS^*n*^) were also reported in some literatures [18–20]. However, identified alkaloids are uneasy using LC/MS due to no available mass spectral database on electrospray ionization (ESI). GC/MS is one of the highest sensitivity analysis techniques, whose limit of detection can easily reach picogram (10^−12^) and provide available standard mass spectral data library [21–23].

In this paper, the processed RC was employed as sample to investigate whether the alkaloids content and composition were changed. The crude RC was processed according to the standard method of Chinese Pharmacopoeia compiled by Pharmacopoeia Commission of Ministry of the Health of China as processed RC sample [24]. The processed RC samples were extracted in Soxhlet extractor with methanol, and the crude RC sample, as control, was extracted with two different solvents, methanol and acetone. A comparison of the differences and similarities between the processed RC and the crude RC on the alkaloids was performed for both qualitative and semiquantitative analyses.

## 2. Experiment 

### 2.1. Reagents

The plant materials were provided from Traditional Chinese Medicine Institute of Heibei University and authenticated by pharmacist Xianmao Liang. A voucher specimen has been deposited in our laboratory. The chemicals purchased from a local chemical company (Hua Xin, Baoding, China) were of analytical reagent grade and included methanol (MeOH), acetone (Ac), dichloromethane, and sodium hydroxide. Acetic acid and hydrochloric acid (12 mol/L) were also purchased from the local chemical company. The water used in the experiments was doubly distilled and prepared in our laboratory.

### 2.2. Processing RC and Extraction

The processed RC was carried out according to the method described in Chinese Pharmacopoeia. Briefly, 10 g of RC was immersed in a beaker (50 mL) with acetic acid solution (30 mL, 36%). The solution was heated until dryness. Then the processed RC was extracted in Soxhlet extractor with 150 mL of MeOH/H_2_O (4/1; V/V) until the extracting solution was colorless. After getting rid of the methanol by water bathing, the residual extracting solution (30 mL) was added to isopycnic HCl solution (12 mol/L). The mixture (60 mL) was refluxed for 1 h and extracted with dichloromethane (20 mL × 3) in a separatory funnel to be degreased after cooling to room temperature. The degreased solution (60 mL) was filtered after adjusting the pH to 8 with NaOH. The filtrate (60 mL) was extracted with dichloromethane (20 mL × 3). The organic phase was collected in a conical flask. 10 mL of the organic phase was transferred to a tube (10 mL) and evaporated to dryness under a N_2_ stream. The residue was dissolved with 1.0 mL of acetone, marked S1, and then stored in −20°C for further GC/MS analysis.

The extractions of the crude RC with methanol and acetone were the same as described above except that the drug had not undergone processing. The methanol and acetone extracts of RC were marked S2 and S3, respectively, and stored in −20°C for further GC/MS analysis.

### 2.3. GC/MS Analysis

GC/MS was performed on an Agilent 7890A GC Plus equipped with an 5975C mass/selective detector (Agilent Technologies). A fuse silica capillary column, HP-5-MS, with 5%-phenyl methylpolysiloxane as no-polar stationary phase (30 m × 0.25 mm i.d. × 0.25 mm film thickness, Agilent Technologies) was utilized for analysis of alkaloids obtained from the processed RC and the crude RC. The injection port temperature was 260°C. The column temperature programme started at 40°C upon injection. The temperature was increased at a rate of 40°C/min to 110°C and then at a rate of 8°C/min to 260°C and held there for 10 min. Purified helium gas at a flow rate of 1 mL/min was used as the GC carrier gas in constant flow fashion. Each sample (1 *μ*L) was injected into the column using a 60 : 1 split injection and solvent delay time was 3 min. The mass spectrometer was operated in the electron impact (EI) mode with an electron energy of 70 eV; ion source temperature, 230°C; quadrupole temperature, 150°C; mass range,* m/z* 38–550; scan rate, 0.25 s/scan; EM voltage, 1423 V; and the GC/MS transfer line was set to 280°C.

## 3. Results and Discussion 

### 3.1. Chromatographic Separation

The processed RC sample was claybank via extraction in Soxhlet extractor and the crude RC samples were faint yellow. An important feature is the cleanup of samples prior to GC/MS analysis because the presence of matrix interference leads to ion suppression or enhancement in many cases [25, 26]. After degreasing, alkaloids were enriched and most micromolecules were wiped off.

**Figure 1:**
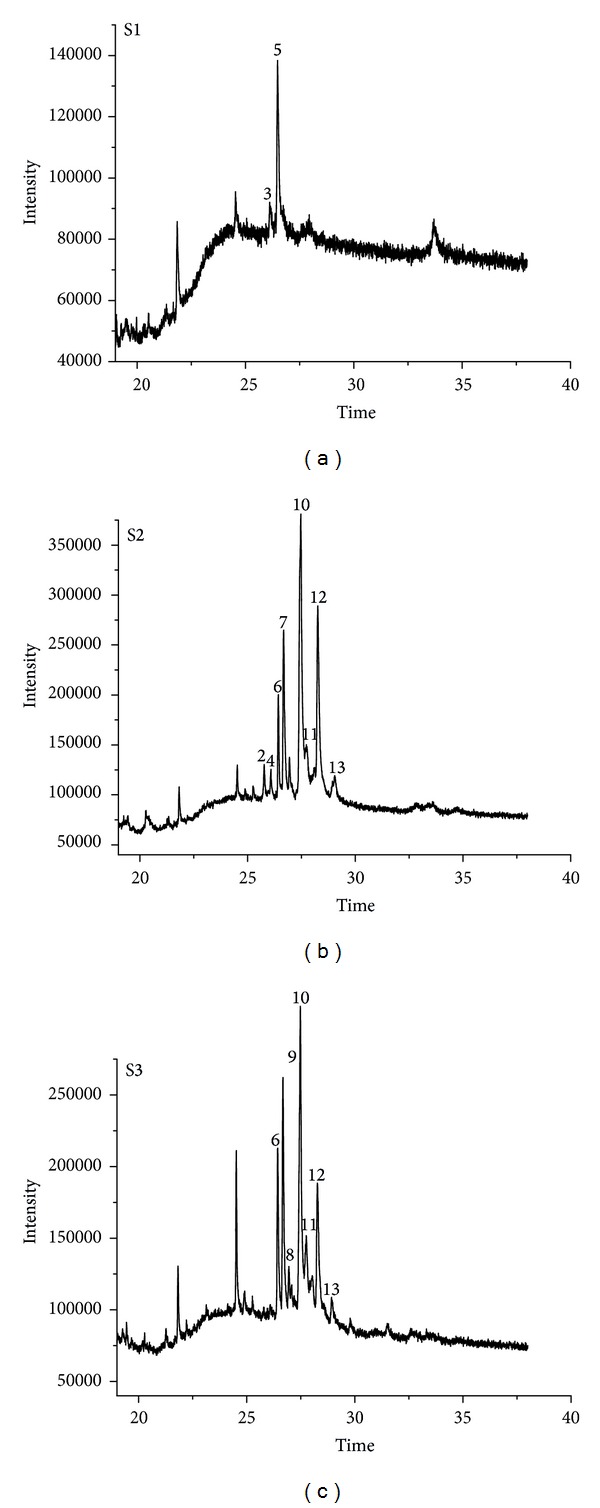
Total ion current chromatograms of RC. S1: processed RC sample extracted with methanol, S2: crude RC sample extracted with methanol, and S3: crude RC sample extracted with acetone. (a) The total chromatogram and (b) the enlarged chromatogram after 19 min. The peak number is consistent with Table 1. 4# and 5# (+)-corydaline. Column: HP-5-MS column, injector temperature: 260°C, GC oven temperature program: 40°C (does not hold), at 40°C/min to 110°C (does not hold), at 8°C/min to 260°C (holds for 10 min).

Figure 1 was the chromatograms of three samples after 19 min. The chromatographic distillate components before 19 min (not shown in Figure 1) are the constituents with low boiling point like alkane, ester, alcohol, ketone, and fatty acid, which are the residual in the degreased process. The alkaloids, however, with higher boiling points and lower vapor pressures were eluted after 19 min. As Figure 1 showed, the components of S1 eluted after 19 min are less than those of S2 and S3 obviously.

More than ten peaks were eluted after 19 min from the crude samples S2 and S3. The retention times of the peaks were the same but the heights of peaks were not equal due to the different concentration between S2 and S3, whereas there were only a few peaks eluted from S1. The processed sample S1 had obvious differences to the crude samples S2 and S3, which indicated that the constituents with high boiling point eluted after 19 min were related more to the processing of the sample rather than to the extraction solvent, which led to content changes and chemical rearrangements of alkaloid components.

### 3.2. Identification of Alkaloids

**Figure 2:**
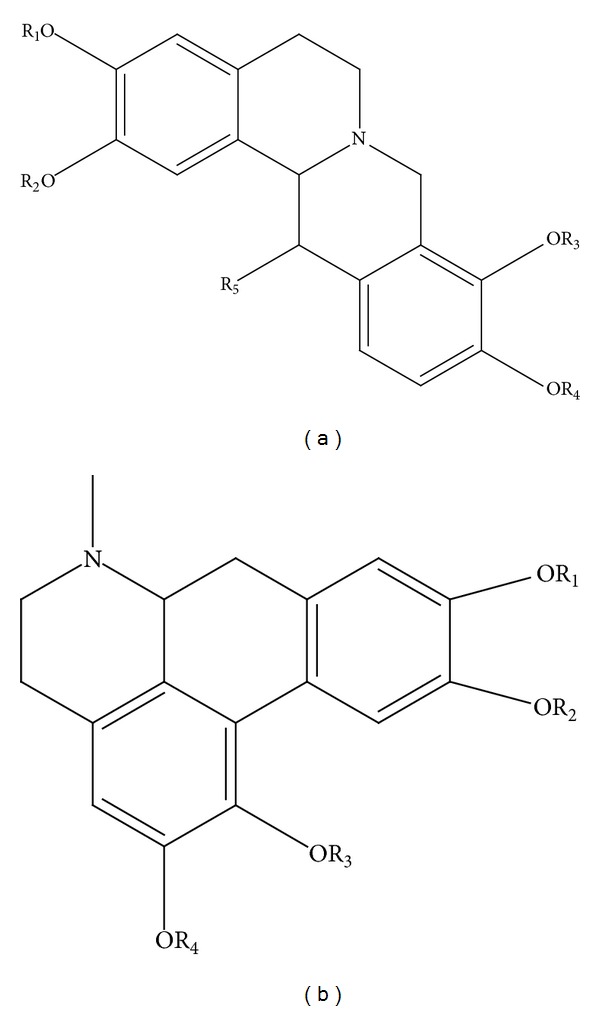
Two foundation formations of alkaloids in Rhizoma Corydalis. (a) Corydaline: R_1_=R_2_=R_3_=R_4_=CH_3_. Thalictricavine: R_1_ + R_2_ = –CH_2_–; R_3_=R_4_=CH_3_.

The identifications of the alkaloid components were based on computer matching of their mass spectral fragmentation patterns with those stored in the spectrometer database using the National Institute of Standards and Technology Mass Spectral Database (NIST-MS, 2008) and artificial interpreting of their fragmentation patterns.  Table 1 showed that a total of 13 kinds of alkaloids including corydaline, glaucine, canadine, and protopine were identified from the crude and processed RC samples. They have two backbone structures to constitute two series of alkaloids (Figure 2). Among them, there were two chromatographic peaks (retention times 20.288 and 28.257) with extremely similar mass spectral fragmentation, hence with the same searching result. The latter peak (28.257) with higher match degree was identified as the search result Isoquino [2.1-b] isoquinoline, 4b,5,10,10a,11,12-hexahydro-2-hydroxy-3,8,9-trismethoxy-, and the former peak was identified as isomer of the latter.

**Figure 3:**
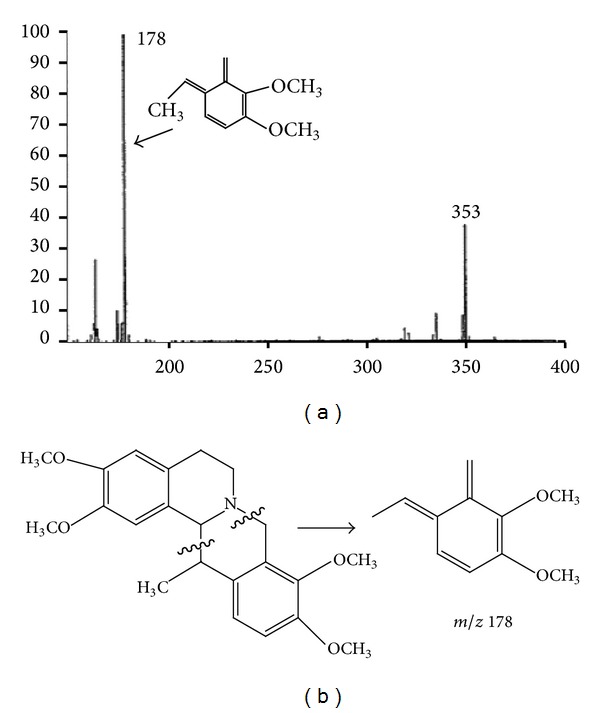
The mass spectrum (EI) of thalictricavine and fragmentation pathway of (+)-corydaline. (a) The mass spectrum of thalictricavine was performed by Rueffer et al.

**Figure 4:**
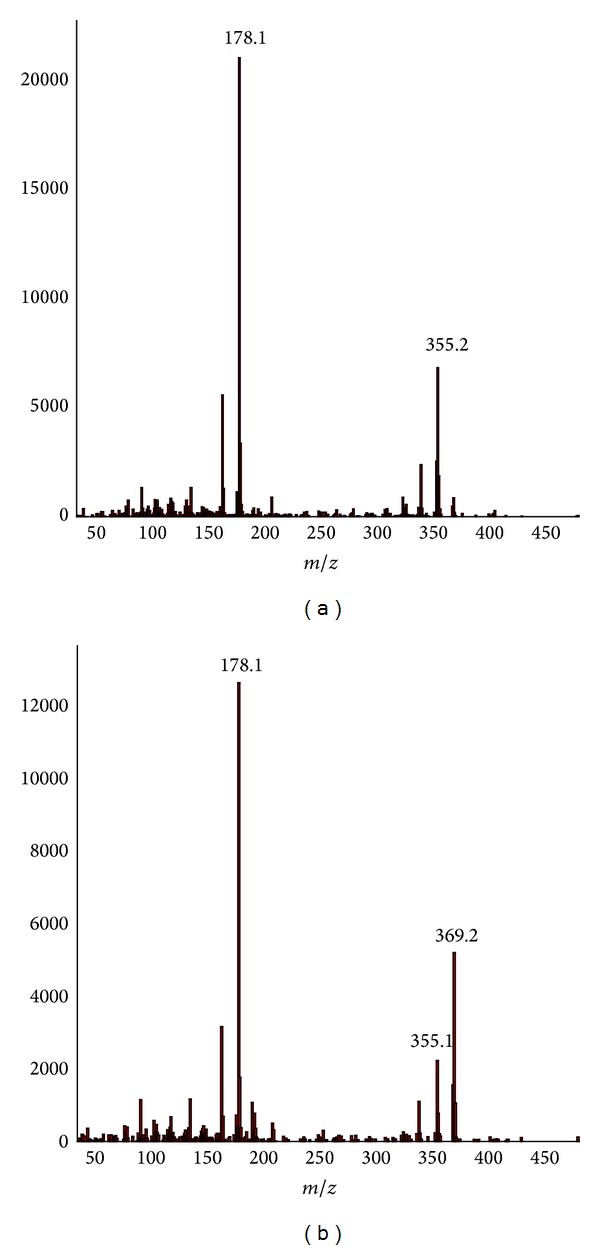
Mass spectrum (EI) of peak of P_4_ (a) and P_5_ (b).

There was no matching with corydaline in Mass Spectral Database (NIST-MS, 2008). The mass spectral data of corydaline were mostly carried out using ESI model due to the poor volatilization of corydaline. With rare fragment ion, the ESI mass spectrum was not equipped with mass spectral database. Hence, ESI mass spectral data was not identified through mass spectral library search. Fortunately, Rueffer et al. [12] had performed the study to probe into a biosynthesis path of these alkaloids using C13 markers, in which themass spectrum ofthalictricavine, very similar to corydaline, was provided using EI model (Figure 3(a)). It indicated that the based fragment ion was* m/z* 178 (base peak) which was cleaved from piperidine (Figure 3(b)).  Figure 4 was the mass spectrum (EI) of chromatographic peak P_4_ (26.426) and P_5 _(26.485). As Figure 4 showed that the base peak of mass spectrum for both P_4_ and P_5 _is* m/z* 178 and the second base peaks are different, P_4_ is* m/z* 355, not the molecule ion, and P_5_ is* m/z* 369, the molecule ion. The compound of P4 was identified as corydaline according to the research by Rueffer et al. [12]. And the P_5_ was identified as conformational isomer of corydaline. This outcome was backed not only by mass spectral data but also by the retention time. The difference of retention time between two peaks was about 3.6 s. It indicated that conformational change happened with corydaline when the RC was processed. A simple acid bath of RC was capable of the conformation conversion of corydaline, which illustrated that the threshold battery of conformation conversion for corydaline was low; and the traditional processing herbal medicine procedure was efficient in reducing their toxicity.

### 3.3. Effect of Different Solvents on Extraction of Alkaloid

Although GC/MS total ion chromatograms (after 19 min) of crude CR samples S2 and S3 were similar to each other, the relative content of component was different.  Table 2 was the relative contents of alkaloids in the crude RC samples S2 and S3. The contents of eluted alkaloids in S2 were higher than those in S3 except 4, 6, and 11# peaks. It suggested that extraction solvent was dependent on the target alkaloids. The most relative content component in S2 and S3 was 10# peak, which was not fully separated and contained two components. It indicated that the chromatographic behavior of these components was very similar. The different oven programs were performed to improve their separation, but the outcome was not as expected.

## 4. Conclusion

The crude RC and processed RC were qualitatively and semiquantitatively analyzed using gas chromatography-mass spectrometry (GC/MS) method. The totals of 13 kinds of alkaloids were identified, in which 11 kinds of alkaloids were from the crude RC sample and two were from the processed RC sample. The 13 kinds of alkaloids are all from two backbone structures forming two series of alkaloids. The extraction of alkaloids was related a little to the solvent used. Most of the alkaloids in crude RC were not detected and the corydaline had been changed in conformation after the RC had undergone processing, which provided support for the conclusion of reducing toxicity in the traditional drugs treatment process.

## Figures and Tables

**Table 1:** Alkaloids identified from processed RC and RC by mass spectrum.

Peaks	Retention time	Compounds	Match degree	Sample source
1	20.288	4b,5,10,10a,11,-12-Hexahydro-3-hydroxy-2,8,9-tri(methoxy) Isoquino [2.1-b] isoquinoline	90	②
2	25.771	N-Methyllaurotetanine	81	②
3	26.158	Glaucine	78	
4	26.420	(+)-Corydaline 178 355 369	—	②, ③
5	26.485	(+)-Corydaline 178 369	—	①
6	26.668	(13S.13aR)-2,3-Dioxhydryl-9,13-methyl-10-ethyl-6,8.13,13a-tetrahydro-5H-isoquinolino[2,1-b]isoquinoline R_1_=R_2_=H, R_3_=CH_3_, R_4_=C_2_H_5_, R_5_=CH_3_ 192, 355	—	②
7	26.936	(13S.13aR)-2,3-(Methylenedioxy)-9,10-dimethoxy-13-methyl- 6,8.13,13a-tetrahydro-5H-isoquinolino[2,1-b]isoquinoline R_1_ + R_2_=–CH_2_–, R_3_=R_4_=R_5_=CH_3_ 178 355	—	②
8	27.076	(+)-Canadine C_20_H_21_O_4_N	76	③
9	27.425	Protopine	90	③
10	27.468	6H-Dibenzo[a,g]quinolizine, 5,8,13,13a-tetrahydro-2,3,10,11-tetramethoxy R_1_=R_2_=R_3_=R_4_=CH_3_, R_5_=H 164, 192 355	84	②, ③
11	27.741	6H-Dibenzo[a,g]quinolizine, 5,8,13,13a-tetrahydro-2,3,9,10- tetramethoxy R_1_=R_2_=R_3_=R_4_=CH_3_, R_5_=H 164, 192 355	83	②, ③
12	28.257	Isoquino [2.1-b] isoquinoline, 4b,5,10,10a,11,12-hexahydro-2- hydroxy-3,8,9-trismethoxy-C_20_H_23_O_4_N 341	92	②, ③
13	29.041	Isocorydine C_20_H_23_O_4_N 341	64	②

“—”: identified by artificial analysis.

Sample source: ① the processed Rhizoma Corydalis sample extracted with methanol, ② the crude Rhizoma Corydalis sample extracted with methanol, and ③ the crude Rhizoma Corydalis sample extracted with acetone.

**Table 2:** The relative contents of alkaloids in the crude RC samples S2 and S3.

Alkaloids and retention time	Relative contents (S2, %)	Relative contents (S3, %)
1^#^ (20.288)	1.36	0.61
2^#^ (25.771)	6.21	4.31
4^#^ (26.420)	7.61	13.15
6^#^ (26.668)	12.64	16.05
7^#^ (26.936)	5.63	5.03
8^#^ (27.076)	—	3.82
10^#^ (27.468)	30.16	26.18
11^#^ (27.741)	7.83	10.38
12^#^ (28.257)	22.12	16.36
13^#^ (29.041)	6.43	4.10

The numbers of alkaloids (*X* ^#^) were consistent with those in Table 1.
